# Perfect Dual-Band Absorber Based on Plasmonic Effect with the Cross-Hair/Nanorod Combination

**DOI:** 10.3390/nano10030493

**Published:** 2020-03-09

**Authors:** Yuan-Fong Chou Chau, Chung-Ting Chou Chao, Hung Ji Huang, Muhammad Raziq Rahimi Kooh, N. T. R. N. Kumara, Chee Ming Lim, Hai-Pang Chiang

**Affiliations:** 1 Centre for Advanced Material and Energy Sciences, Universiti Brunei Darussalam, Tungku Link, Gadong BE1410, Brunei; chernyuan@hotmail.com (M.R.R.K.); roshan.kumara@ubd.edu.bn (N.T.R.N.K.); cheeming.lim@ubd.edu.bn (C.M.L.); 2 Department of Optoelectronics and Materials Technology, National Taiwan Ocean University, Keelung 20224, Taiwan; suyang191@gmail.com; 3 Taiwan Instrument Research Institute, National Applied Research Laboratories, Hsinchu 300, Taiwan; hjhuang@narlabs.org.tw; 4 Institute of Physics, Academia Sinica, Taipei 115, Taiwan

**Keywords:** plasmonic effect, localized plasmon modes, dual-band plasmonic perfect absorber, absorptance peaks, plasmonic sensor

## Abstract

Plasmonic effect using a cross-hair can convey strongly localized surface plasmon modes among the separated composite nanostructures. Compared to its counterpart without the cross-hair, this characteristic has the remarkable merit of enhancing absorptance at resonance and can make the structure carry out a dual-band plasmonic perfect absorber (PPA). In this paper, we propose and design a novel dual-band PPA with a gathering of four metal-shell nanorods using a cross-hair operating at visible and near-infrared regions. Two absorptance peaks at 1050 nm and 750 nm with maximal absorptance of 99.59% and 99.89% for modes 1 and 2, respectively, are detected. High sensitivity of 1200 nm refractive unit (1/RIU), figure of merit of 26.67 and Q factor of 23.33 are acquired, which are very remarkable compared with the other PPAs. In addition, the absorptance in mode 1 is about nine times compared to its counterpart without the cross-hair. The proposed structure gives a novel inspiration for the design of a tunable dual-band PPA, which can be exploited for plasmonic sensor and other nanophotonic devices.

## 1. Introduction

With the ability of efficaciously transforming electromagnetic (EM) waves from free space into the sub-wavelength scale, plasmonic nanomaterials disclose a broad range of applications in nanophotonics [1,2,3,4,5,6]. Metal nanoparticles (MNPs) with the behavior of surface plasmon polaritons (SPPs) and resonant couplings lead to EM wave confinement that can be applied in nanophotonic and optoelectronic fields [7,8,9,10,11,12]. Surface plasmon resonance (SPR) is an extraordinary phenomenon, which originates from coupling an EM wave with collective electron oscillations between the interface of an MNP and dielectric [13,14,15,16,17]. In the same manner, the gap plasmon resonance (GPR), cavity plasmon resonance (CPR) and lattice plasmon resonance (LPR) phenomena will be generated in composite MNP-dielectric nanostructures when the resonance modes are attributed to gaps, cavities and periodic nanostructure arrays, respectively [18,19,20,21,22,23]. 

A perfect absorber is a subject worth researching and had been substantially developed [24,25,26,27,28]. Recently, much research has attracted attention in perfect absorptance in composite MNP-dielectric nanostructures because of its useful applications in designing nanophotonic devices [29,30]. The rapidly increasing requirement for portable, highly sensitive plasmonic sensors has provided a great benefit to the advancement of plasmonic-based sensing technology. A number of concerns has been focused on the investigation of designing perfect EM wave absorbers, given that the refractive index (RI) in plasmonic sensing design is a potential subject in nanophotonics [31,32,33,34,35,36]. The SPR properties corresponding to a tunable refractive index and resonance mode occurring in MNP-dielectric nanostructures are attractive for applying in many applications, such as plasmonic sensing [37], photovoltaics [38], spectroscopy [39], photodetection [40], recording [41], surface-enhanced Raman spectroscopy (SERS) [42], optical filters [43] and other adaptive photonic devices [44], which require high-performance perfect absorbers [45].

Metal nanorods can be used as plasmonic material for improved spectroscopy both in visible and near-infrared spectra [46]. Particularly, the distinctive morphology results in higher EM wave enhancement and localization compared to conventional plasmonic materials [47]. This characteristic can be used to realize ultra-high sensitivity to the variations of the surrounding medium, making it an interesting device for the design of sensors and biosensors [48]. The valuable design of plasmonic perfect absorbers (PPAs) is highly desirable for easily dropped analytes or gases in voids and bond with the sensing medium, higher sensitivity and dual-band spectra response [49,50,51]. When the incident EM wave impinges on the PPA surface, both the amplitude of reflectance and transmittance are declined to zero, which indicates that the EM wave is completely absorbed by the material with unity absorptance. Recently, several PPAs based on all-metal or composite MNP-dielectric nanorod structures for refractive index sensing application have been designed [52,53]. However, these nanosensors operated only in a single-band, thus restricting the working spectrum, especially, ranging in both visible and near-infrared regions.

It is well-known that with two or several metal nanorods placed closely to each other, bonding and antibonding modes can be induced, and these modes originate from the behaviors of the hybridization plasmon of SPR on the metal surface and GPR among MNPs [54]. A periodic array of metal-shell nanorods can act as plasmonic sensors due to its strong cavity enhancement property (i.e., CPR) [55] and lattice resonance characteristic (i.e., LPR) [56]. In our previous work [24], a coupled Ag-shell/dielectric-core (ASDC) nanorod structure with a small gap distance (*g* = 20 nm) and single band of unity absorptance was proposed. The drawback of the proposed ASDC structure is a lower absorptance due to the smaller gap and cavity coupling among the metal-shell nanorods when the gap distance is increased (e.g., *g* > 40 nm [57]). This implies that the hybridization of resonance coupling of SPR, GPR, CPR and LPR modes can be simultaneously acquired in a periodic array of metal-shell nanorods connected with the bridges between them even if a larger gap distance exists among the nanorods (e.g., *g* = 80 nm). A plasmonic effect, which is formed by a cross-hair among the composite MNP-dielectric nanostructures, can act as the bridge of the four above-mentioned modes in a photonic–plasmon system. Due to its plasmonic property, it is possible to tightly combine the EM wave at a deep sub-wavelength nanoscale to significantly enhance light localization and absorptance [58,59]. The cross-hair can be regarded as a functional bridge that effectively binds and traps the scattering and reflecting EM waves, thus achieving enhanced absorptance and even unity absorptance of the plasmonic nanostructure. This motivates us to study how the plasmonic effect affects the electric field, magnetic field, energy flows, surface charge density and resonance modes in the composite MNP-dielectric nanostructures, which, up to now, have not been addressed in detail and are still not very clearly understood in previous studies.

Inspired by the above-mentioned crucial issue, in this work we propose and design a tunable perfect dual-band absorber with an assembly of four metal-shell nanorods connected by a cross-hair operating in visible and near-infrared regions using the three-dimensional (3-D) finite element method (FEM) [60]. We demonstrate strongly enhanced EM wave distribution on the gaps, metal-shell cavities, metal and cross-hair surfaces, which can simultaneously support the SPR, GPR, CPR and LPR modes and attain dual-band unity absorptance with high tunability and sensitivity. The impact of geometrical parameters on the absorbance spectrum of the design, EM wave confinement mechanism inside the proposed PPA structure and superiority of the cross-hair over other metals are investigated. It is found that the connected cross-hair among the separated metal nanostructures leads to a unique plasmonic effect, which can significantly enhance EM wave and localized plasmon modes in the proposed PPA. Compared to its counterpart without the cross-hair, this characteristic has the remarkable merit of enhancing absorptance at resonance and can make the structure carry out perfect dual-band absorptance. The physical origin of dual-band perfect absorption peaks is related to the SPR, GPR, CPR and LPR modes that simultaneously occur in the proposed PPA based on the plasmonic effect arising from the cross-hair/nanorod combination. We believe that the proposed structure gives a novel inspiration for the design of a tunable dual-band absorber, which can be applied to plasmonic sensors and other nanophotonic devices.

## 2. Structure Design and Simulation Method

**Figure 1:**
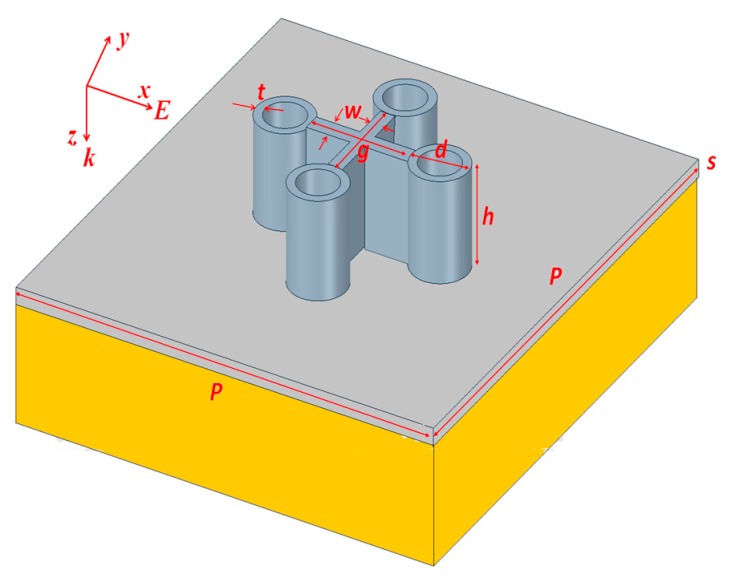
Schematic plots of a unit cell of the proposed plasmonic perfect absorber (PPA).

The proposed three-dimensional PPA structure is periodic, and the model system of a unit cell is illustrated in Figure 1, which includes four silver-shell nanorods with air cores (*n* = 1.0) connected by two silver-made crossing bridges with equal-length arms (i.e., the cross-hair), which were used for simulation employing the 3-D FEM. The origin ((*x*,*y*,*z*) = (0,0,0)) of the coordinate system is located in the middle plane of the unit cell. The designed structure array is periodically configured on the surface of a silver layer placed on the glass substrate (*s* = 100 nm in thickness) with a period (*P*). Perfect matched layers (PMLs) were used along *z*-axes to avoid any reflection from boundaries, and periodic boundary conditions were used in *x*- and *y*-axis to replicate an infinite array of the unit-cell structure, respectively. The domain above the PPA structure was set to be air (*n* = 1.0). The dielectric constant of silver and glass substrate was obtained from [61,62], respectively. In the inset of Figure 1, the other structural parameters of *P* (period), *w* (the widths of the cross-hair), *h* (the height of silver-shell nanorod), *d* (the outer diameter of silver-shell nanorod), *g* (length of silver cross-hair, i.e., gap distance between two opposite nanorods) and *t* (thickness of silver-shell nanorod) were set to be *P* = 470 nm, *w* = 30 nm, *h* = 150 nm, *d* = 80 nm, *g* = 80 nm and *t* = 10 nm, respectively, unless specified otherwise. A normal incidence plane incident EM wave with transverse polarization was performed by fixing at |E_0_| = 1 V/m. It can be expected that the optical spectrum is nearly unchanged under normal incidence when varying the polarization for both *x*- and *y*- polarizations due to the high rotation symmetry of the proposed unit-cell structure, which will be a great benefit to design a PPA in practical applications. The silver-shell nanorods in the proposed structure can be considered as a monopole antenna where SPRs are excited at the metal–air interface. The absorptance (A) can be calculated by A(*ω*) = 1 – *R*(*ω*) – *T*(*ω*), where *R*(*ω*) and *T*(*ω*) denote the reflectance and transmittance, respectively, as functions of frequency (*ω*). The sensitivity (*S*), quality factor (*Q* factor) and figure of merit (*FOM*) can be defined as *S* = Δλ/Δ*n*, *Q* = λ_res_/FWHM and *FOM* = *S*/*FWHM*, respectively, where Δλ is the shift of resonant peak wavelength of absorptance, λ_res_ is the resonant wavelength, Δ*n* is the refractive index difference and FWHM is the full width at half maximum bandwidth of the resonance absorptance wavelength.

Thanks to the fast progress in the nanofabrication technology, an advantage of using an array of the proposed structure on the top surface is that it is compatible with all current nanofabrication technologies such as a manufacturing based on secondary electron lithography generated by ion beam milling [63,64,65,66,67,68] and other fabricating procedures [69,70]. This could easily result in the possible manufacture of the proposed PPA.

## 3. Results and Discussion

**Figure 2:**
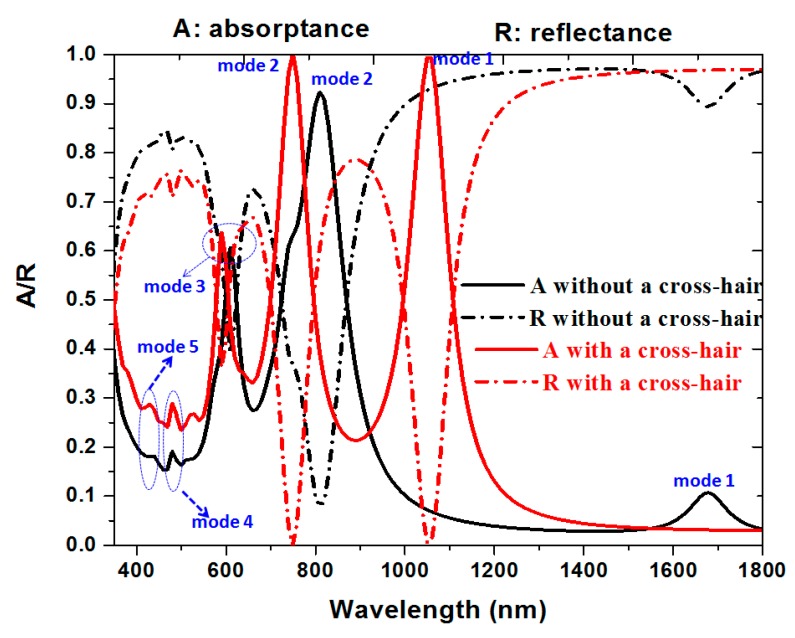
Absorptance (A) and reflectance (R) spectra of the proposed PPA (red and pink lines). The results for its counterpart without a cross-hair (black and blue lines) are included for comparisons.

Figure 2 shows the absorptance (A) and reflectance (R) spectra of the proposed PPA (red and pink lines). The results for its counterpart without the cross-hair (black and blue lines) are included for comparison. Using the structural parameters as mentioned in Section 2, nearly no transmittance of incident EM wave is revealed in the near-infrared region for the proposed PPA at resonance wavelengths. At the ultra-visible and visible regions, the material properties become the governing factor in deciding the entire reflectance from the structure. The difference of resonance peaks/dips between two cases can be attributed to that of the dissimilar hybridization plasmon mode that occurs in the composite MNP-dielectric nanostructures. This comes from the fact that the optical characteristics of proposed PPA support excellent impedance matching from air to the metal–air composite; therefore, such an elevated absorptance is achieved in visible and near-infrared regions. When the proposed PPA is considered from a monopole antenna standpoint, multiple SPPs at different resonance wavelengths are induced at the metal–air interface. It is shown that there are five resonance modes (termed as mode 1–5 as indicated in Figure 1), and these resonance modes attribute to the different resonance conditions among the incident EM wave, gaps, metal-shells and a cross-hair. When four structures of silver-shell nanorods in a unit cell are connected by a cross-hair, two nearly perfect absorptance modes will be excited by their interaction. These interactions of plasmonic effect can further increase the resonance absorption of the proposed PPA. Therefore, we can substantiate that mode 2 is mainly corresponded to the interaction between the incident EM wave, gaps among nanometals and cavity parts in the silver-shell nanorods (Fabry–Perot resonance), which is related to the localized SPR, gap plasmon resonance (i.e., GPR) and cavity plasmon resonance (i.e., CPR). The excitation of mode 1 is mainly excited by the incident EM wave, gaps, the cavities in silver-shell nanorods, lattice plasmon resonances (LPR) and the cross-hair. The strong EM wave distribution near the proposed PPA is derived from hybridization of localized SPR, GPR, CPR and LPR modes due to the plasmonic effect being introduced in the proposed photonic–plasmon resonance system using a cross-hair.

The absorptance peaks of mode 1 (at *λ*_res_ = 1050 nm) and mode 2 (at *λ*_res_ = 750 nm) with 99.59% and 99.89% can be acquired, revealing a dual-band perfect absorptance. In the case without a cross-hair, the absorptance peaks show less values, i.e., 10.68% at *λ*_res_ = 1680 nm for mode 1 and 92.34% at λ_res_ = 810 nm for mode 2, respectively. It is worth noting that the absorptance of the proposed PPA in mode 1 is about nine times compared with the case without the cross-hair. This is due to the fact that the contribution originated from the plasmonic effect of a cross-hair/nanorod combination, which shows a remarkable influence on the plasmon resonance in the proposed PPA.

**Figure 3:**
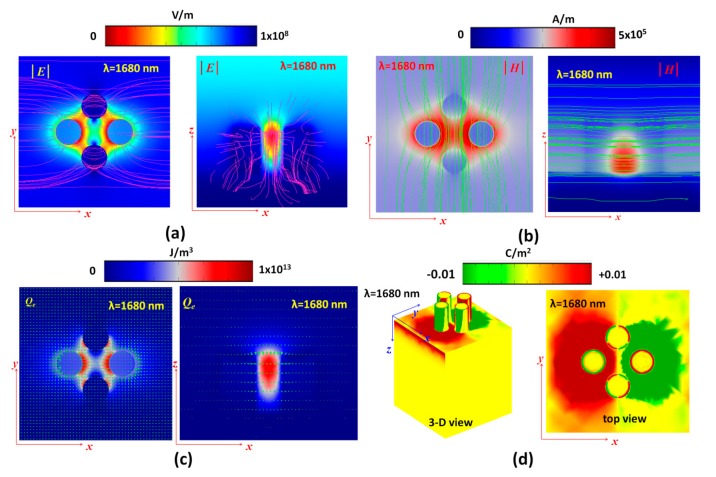
(**a**) Electric field intensity (|E|, V/m) with electromotive force lines (pink lines), (**b**) magnetic field intensity (|H|, A/m) with magnetic fluxes (green lines), (**c**) energy density (J/m3) with power flow (green arrows) and (**d**) surface charge density distributions (3-D and top views) of the cases without the cross-hair at λ_res_ = 1680 nm, where |E| and |H| field intensities and energy density are measured at the bottom plane of silver-shell nanorods for the x–y plane and at the middle plane of silver-shell nanorod for the *x*–*z* plane.

**Figure 4:**
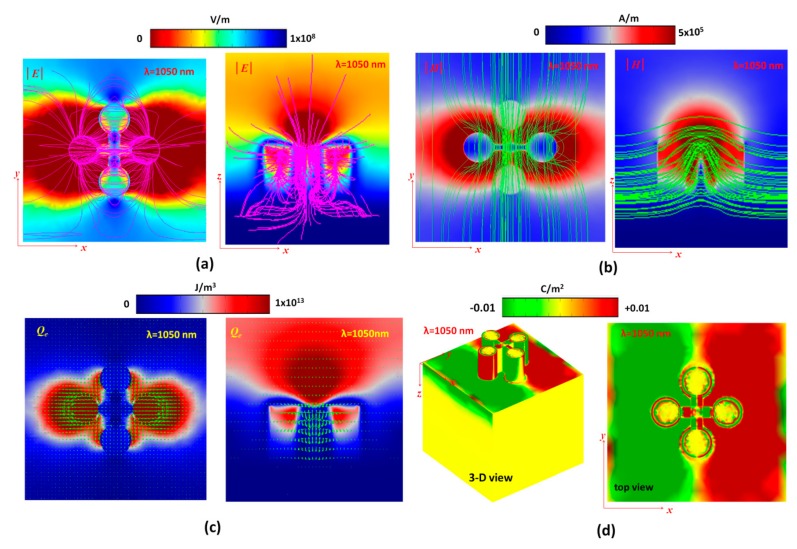
(**a**) Electric field intensity (|E|, V/m) with electromotive force lines (pink lines), (**b**) magnetic field intensity (|H|, A/m) with magnetic fluxes (green lines), (**c**) energy density (J/m^3^) with power flow (green arrows) and (**d**) surface charge density distributions (3-D and top views) of the cases with the cross-hair at *λ*_res_ = 1050 nm, where |E| and |H| field intensities and energy density are measured at the bottom plane of silver-shell nanorods for the *x*–*y* plane and at the middle plane of silver-shell nanorod for the *x*–*z* plane.

To better reveal the mechanism of the resonance modes in the proposed PPA, the electric field intensity (|E|, V/m) with electromotive force lines (pink lines), magnetic field intensity (|H|, A/m) with magnetic fluxes (green lines), energy density (J/m^3^) with power flow arrows (green arrows) and surface charge density distributions (3-D and top views) of the cases without (at *λ*_res_ = 1680 nm) and with (at *λ*_res_ = 1050 nm) the cross-hair at mode 1 are shown in Figure 3a–d and Figure 4a–d, respectively. As mentioned previously, perfect absorptance stems mainly from the localized SPR, GPR, CPR and LPR in the proposed PPA structure and excitation of the fundamental dipole coupling mode. Different incident wavelength of EM wave interacts with the metal–air composite nanostructure leads to different surface charge distribution. The surface current on the metal surface could be enhanced by the positive–negative charge pairs, and they induced the electromotive force lines and magnetic fluxes. As seen in Figure 3a,b and Figure 4a,b, it is evident in both cases that the maximum |E| and |H| are strongly confined at the interface of air/MNP and the gaps between MNPs, indicating that there is excitation of localized SPR and GPR here. As can be observed, the proposed PPA possesses stronger EM wave distributions and denser electromotive force lines, magnetic flux lines and power flow arrows (see Figure 4a–c) than its counterpart without a cross-hair (see Figure 3a–c), since the plasmonic effect induces strong in-plane and out-plane EM wave coupling across the gaps, edges and the cross-hair. The electric field and magnetic fields in both cases at the resonant wavelength are driven by the incident EM wave and oscillate in time. The electric field is concentrated around the gaps and bottom circumference of the silver-shell nanorods, while the magnetic field is concentrated directly above the top surface and adjacent to the side walls of the silver-shell nanorods. Note that the electric field has a larger out-plane spatial distribution than the magnetic field in the proposed PPA, as the electric field does not decline as sharply with the distance away from the surface of the MNPs.

In Figure 3c, we can see that EM energy density with power arrows is mostly absorbed in the gap and at the circumference of the top surface of the silver-shell nanorods. As seen in Figure 4c, optical energy is mostly confined and harnessed at the top edges of the silver-shell nanorods while this energy is localized around the gap and side walls of the silver-shell nanorods. This power absorption by the proposed PPA causes a dip in the reflectance spectrum, as no power is reflected back at the resonance wavelength. This fundamental mode remarkably exists for the case with the cross-hair in the proposed PPA. 

The mechanism of |E| and |H| profiles can be also explained by the surface charge density (Coulomb/m^2^) as shown in the Figure 3d and Figure 4d. From the surface charge density distribution diagrams, negative and positive electric charges are mainly distributed on the opposite ends of the 3-D resonator structure, revealing that a strong electric dipole resonance mode is excited under the incident EM wave illuminations. The in-plane EM wave coupling among the proposed PPA leads to the “hot spots” in the gap regions (i.e., GPR), which also result in the surface “hot spots” on the cross-hair due to the surface charge distributions (see Figure 4d). Compared to the case without the cross-hair (see Figure 3d), the cross-hair could provide an additional path, which induces more surface charge pairs on the metal surface and enhances the coupling of dipole resonance. A Fabry–Perot cavity arises along the *z* axis in the metal-shell nanorods, where positive–negative charge pairs on the surface of metal-shell nanorods are observed. The resonance mode is described by its structurally symmetrical charge distribution where the proposed PPA has net positive–negative charges on the surface of metal-shells and the cross-hair. The distribution of surface charges in two cases is reversed at the bottom of the silver layer; meanwhile, the charge distributions on the silver-shell nanorods in two cases are quite different due to the different resonance condition arising from the connected cross-hair. 

Since the capacitance effect of the cavities and gaps among silver-shell nanorods and the inductance effect of the cross-hair becomes strong, the induced electric and magnetic fields among the capacitors and inductances of the proposed PPA structure is stronger [71,72,73,74,75]. This feature is in good agreement with the results obtained from Figure 4. At the resonance condition, energy can be stored in the capacitive form, i.e., a strong electric field parallel to the top surface of the silver-shell nanorods and another strong electric field region at the bottom surfaces of the silver-shell nanorods. The metallic surfaces, including the cross-hair, could contribute a conductive path (or a bridge) for surface charges to flow easily from the top of silver-shell nanorods and the cross-hair into the bottom silver layer and vice versa. This is why the case with a cross-hair has a significant plasmon effect compared to its counterpart without the cross-hair. The electric field, magnetic field, energy density and surface charge distributions of the unit-cell structure indicate that the perfect absorbance is originated from the excitation of the fundamental magnetic and electric dipole resonance modes (i.e., SPR, GPR, CPR and LPR), and the contribution of the plasmonic effect with a cross-hair/nanorod combination.

**Figure 5:**
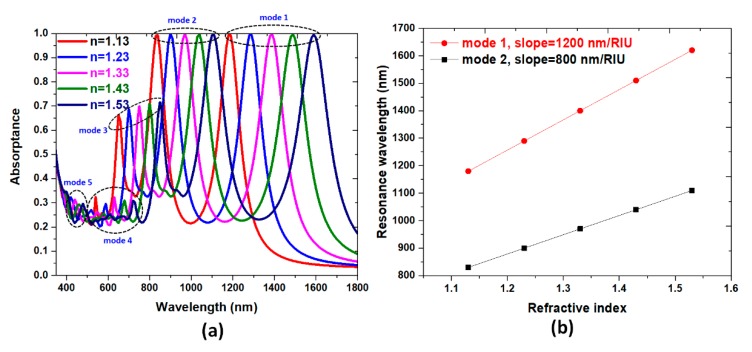
(**a**) The absorptance spectrum of proposed PPA under different ambient refractive indices varied from 1.13 to 1.53 in steps of 0.10. (**b**) The linear relationship of the absorptance spectrum of the proposed PPA versus the variation of surrounding refractive index.

Sensitivity (*S*), figure-of-merit (*FOM*) and *Q* factor are potential parameters for characterizing sensing performance [76,77,78,79], and it is usual to use the perfect absorptance structure of plasmonic sensing. To examine the sensing performance of the proposal PPA, the absorptance spectrum under different ambient refractive indices is inspected when the refractive indices are varied from 1.13 to 1.53 in steps of 0.10. The corresponding absorptance spectra of the proposal PPA with different sensing medium refractive indices are shown in Figure 5a, where a remarkable redshift of absorptance peaks is observed. The reason behind this is when refractive index increases, *n*_eff_ (effective refractive index) of the SPP mode also increases. As a result, the intersection between core mode and SPP mode takes place at a higher wavelength. It can be seen that for modes 1–5, the absorptance peak wavelength has a redshift with the increasing of surrounding RI, and perfect absorptance can be found in mode 1 and 2 simultaneously. The slopes of the linear lines in Figure 5b represent, respectively, the sensitivity of the mode 1 and mode 2. With the calculation, we can obtain *S* = 1200.0 (nm/RIU), *FOM* = 26.67 (1/RIU) and *Q* = 23.3 for mode 1, and *S* = 800.0 (nm/RIU), *FOM* = 20.00 (1/RIU) and *Q* = 21.35 for mode 2, respectively. The advantage of dual band perfect absorptance possesses a broad range of spectrum application (e.g., both in visible and infrared regions) for bulk sensing compared with single band perfect absorptance [24]. The sensing performance of the designed dual-band PPA is very remarkable compared to that of reported literatures [80,81,82,83,84,85,86,87,88,89]. These features provide adequate guidelines for designing high performance plasmonic sensors. 

**Figure 6:**
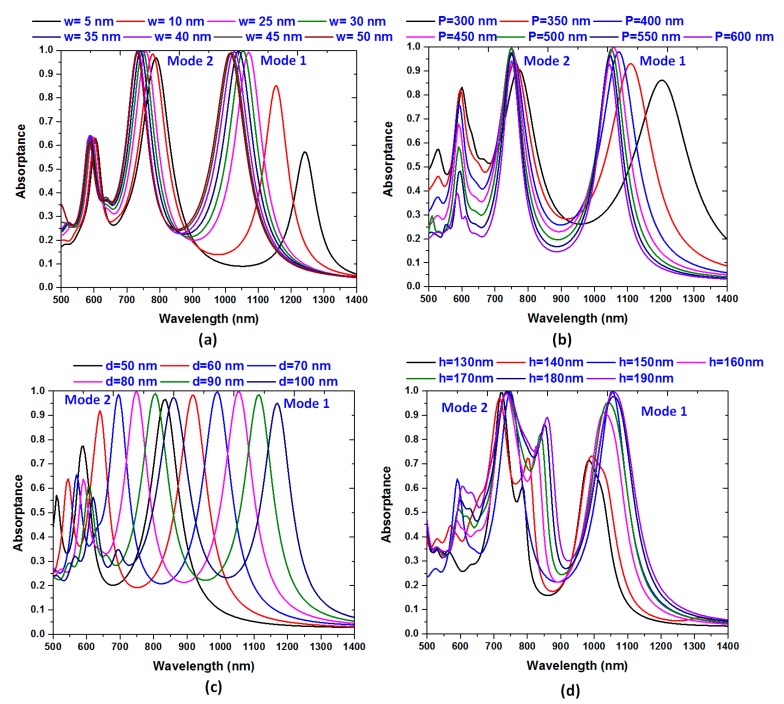
Absorptance spectra of different structural parameters of the proposed PPA, including (**a**) the width of the cross-hair (w), (**b**) period (P), (**c**) outer diameter of the silver-shell nanorod (*d*) and (**d**) height of metal cross-hair (*h*), respectively. The other parameters are kept the same as used in Figure 2.

Finally, we inspect the influence of different structural parameters of the proposed PPA, including the width of the cross-hair (*w*), period (*P*), outer diameter of the silver-shell nanorod (*d*) and height of metal cross-hair (*h*) on the absorptance spectrum. To exam the proposed PPA structure, one parameter is varied at a time, while fixing the other parameters constant (i.e., the same as used in Section 2). As Figure 6a clearly displays for *w* = 5 and 25 nm, the absorptance peaks below 85% are obtained in mode 1, while the absorptance above 95% is acquired in mode 2 due to the lower plasmonic effect arising from the narrower cross-hair. With the increase of *w*, the absorptance peaks of both mode 1 and mode 2 increase and reveal near unity in the range of *w* = 25 to 50 nm, while the absorptance peak bandwidth becomes narrow. As the *w* becomes larger, plasmonic effect is supported with the design (which is robust against manufacturing inaccuracies) and hybridization of SPR, GPR, CPR and LPR modes with the plasmonic effect in proposed PPA, leading to a dual band near unity absorptance. In these cases, the width of cross-hair will dominate the plasmonic effect in the proposed PPA.

Another important parameter is the density of the periodic PPA array, which is *P*. The lattice resonances of proposed PPA array can be manipulated over a broad spectrum by varying the array period. To investigate the influence of the period, *P*, the absorptance spectra for the proposed PPA arrays with *P* values in the range of (300, 350, 400, 450, 500, 550, 600) nm was explored while keeping the other parameters intact (see Figure 6b). When the proposed PPA has a small period, as *P* is set to be *P* = 300 nm and 350 nm, respectively, absorptance is less than 90% and has a significant blue shift. This is because of the destructive interference of plasmon modes among neighboring unit cells. As can be seen that both mode 1 and mode 2 have a slight blue shift, and a near unity magnitude of absorptance as *P* is in the range of 400–500 nm. In fact, the response is mainly originated from the lattice plasmon resonance (i.e., LPR) and GPR. These are in line with our previous findings [90,91]. 

The absorptance spectra for the proposed PPA with varying outer diameter (*d*) of the metal-shell nanorod and height of the cross-hair (*h*) are investigated, as shown in Figure 6c,d, respectively. As it is shown in Figure 6c,d, different cavity sizes toward transverse and vertical axis are illustrated to result in different plasmonic modes that could induce different responses necessitated for the proposed PPA [92,93,94,95]. The cavity being in metal-shell nanorods, the cavity path can support interior plasmon modes of PPA with a localization of the EM wave concentrated in the nanometer scale [96,97,98,99,100,101,102,103]. The absorptance peak red-shifts with the increasing *d* (i.e., increasing the *x*-direction cavity capacity) and *h* (i.e., increasing the *z*-direction cavity capacity). The varying *d* and *h* would generate a variation of the cavity capacity in the proposed PPA and lead to the change of the surface charge distribution on the metal surface, thus forming the strong coupled transverse and vertical CPR modes, which result in the near unity absorptance with proper cavity capacity in the proposed PPA. In Figure 6c,d, the absorptance peak wavelengths red-shift from 850 to 1150 nm for mode 1 and 570 to 825 nm for mode 2 with the increasing *d* in the range of (50, 60, 70, 80, 90 and 100) (Figure 6c), and from 960 to 1070 nm for mode 1 and 700 to 745 nm for mode 2 with the increasing *h* in the range of (130, 140, 150, 160, 170, 180 and 190) (Figure 6d), respectively. The red-shift of the absorptance peak can be caused by the increase in plasmon resonance among each composite nanostructure with the cross-hair [104,105,106,107,108,109]. Additionally, the increase in *d* or the decrease in *h* in the proposed PPA will led to a red-shift in the absorptance peak. There is a maximum at *h* = 150 nm for both the mode 1 and mode 2. The discrepancies in the observed trends in absorptance spectra are attributable to the difference of coupling effect arising from the transverse and vertical CPR with respect to the incident wavelength of EM wave and the length (i.e., *d* and *h*) of nanocavity routes in the proposed PPA. Here, the band linewidth of the coupled photonic–plasmonic resonance is associated with the metal-shell nanorod with a cavity connect by the cross-hair, which is linked to the *Q*-factor because of the modified photonic density of states and hence the modified radiative damping rate. From Figure 6a–d, we observe that the greater the coupling effect on the SPRs, GPRs, CPRs and LPRs, the more absorptance the proposed PPA exhibits. Note that the absorptance spectra of the proposed PPA can be spread in the visible and near-infrared regions by varying outer diameter (*d*) (see Figure 6c). This implies that the plasmon resonance arising from the proposed PPA can be easily tuned by adjusting its geometrical parameters. Anyway, the main advantage of using the plasmonic effect with the cross-hair is the possibility to finely tune the plasmon modes over a broad spectral range, modifying the structural parameters of the array.

For the plasmonic nanorod structure, it is not possible to acquire the same dual band with perfect absorptance by optimizing a design without a cross-hair/nanorod combination due to less coupling among the cavities inside the nanorods and the gaps among the nanorods, i.e., a large gap distance (e.g., *g* = 80 nm) leads to less gap and cavity plasmon resonances. As indicated in [24] (the gap distance of which is set to be *g* = 20 nm), only one perfect absorptance band can be achieved by optimizing the structural parameters, and the absorptance peaks will be dropped rapidly as the gap distance is increased. In real situations, the gap distance among metal-shell nanorods should be given a larger range for robustness in the fabrication process. The proposed PPA structure with the dual band absorptance and high sensitivity is superior to its counterpart without the cross-hair when the gap distance is set to be 80 nm. From the above simulation results, our model depends on the periodicity, the cross-hair dimension, the width and height of the cross-hair and the outer diameter of metal-shell. By varying the above structural parameters, the absorptance peaks can be relocated. At the same time, it can maintain their dual-band characteristics. The optimum parameters for our proposed PPA are determined as *w* = 25~50 nm, *P* = 400~500 nm, *d* = 50~200 nm and *h* = 150~190 nm, respectively, when the dual-band absorptance is required above 90%.

## 4. Conclusions

Summing up, we have proposed a dual-band PPA based on the plasmonic effect, which has a function of confining and conveying EM waves among the composite MNP-dielectric nanostructures with a cross-hair/nanorod combination. Two perfect absorptance peaks appear in the visible and infrared regions. The numerical results show that the absorptance of proposal PPA can be achieved with near unity dual-band absorptance of 99.59% and 99.89% at 1050 nm and 750 nm for mode 1 and 2, respectively. In addition, the absorptance obtained from mode 1 is about nine times compared with its counterpart without the cross-hair. The proposed PPA can be used as a plasmonic sensor for refractive index sensing. With the calculation, we can obtain *S* = 1200.0 (nm/RIU), *FOM* = 26.67 (1/RIU) and *Q* = 23.3 for mode 1, and *S* = 800.0 (nm/RIU), *FOM* = 20.00 (1/RIU) and *Q* = 21.35 for mode 2, respectively, which are on par with the other reported values. The physical origin of dual-band perfect absorptance peaks is related to the SPR, GPR, CPR and LPR modes being simultaneously occurring in the proposed PPA based on the plasmonic effect arising from the cross-hair connected with composite MNP-dielectric nanostructures. We believe that the proposed PPA could offer great potential applications in various biosensing, tunable spectral detecting, plasmonic photocatalysis and other nanophotonic devices.
